# Revealing enterovirus infection in chronic human disorders: An integrated diagnostic approach

**DOI:** 10.1038/s41598-017-04993-y

**Published:** 2017-07-10

**Authors:** Angelo Genoni, Filippo Canducci, Agostino Rossi, Francesco Broccolo, Konstantin Chumakov, Giorgio Bono, Jorge Salerno-Uriarte, Alessandro Salvatoni, Alberto Pugliese, Antonio Toniolo

**Affiliations:** 1 Medical Microbiology, Dept. of Biotechnology and Life Sciences, University of Insubria, Varese, 21100 Italy; 2 Medical Microbiology, Dept. of Medical Sciences, University Milano Bicocca, Milano, 20126 Italy; 3 Office for Vaccines Research and Review, FDA Center for Biologics Evaluation and Research, Silver Spring, MD 20993-0002 USA; 4 Neurology, Dept. of Biotechnology and Life Sciences, University of Insubria, Varese, 21100 Italy; 5 Cardiology, Dept. of Clinical Medicine, University of Insubria, Varese, 21100 Italy; 6 Pediatrics, Dept. of Clinical Medicine, University of Insubria, Varese, 21100 Italy; 7 Diabetes Research Institute, University of Miami Miller School of Medicine, Miami, FL 33136 USA

## Abstract

Enteroviruses (EVs) causing persisting infection are characterized by minimal replication and genetic changes. Typing of these agents may complement disease assessment and shed light on pathogenesis. Here we report an integrated approach for EV detection in human samples that is based on pre-enrichment of virus in cell culture before search for the viral genome and viral antigens. Cases of post-polio syndrome, type 1 diabetes, and chronic cardiomyopathy were investigated. As tissue-based approaches require invasive procedures, information was mainly gleaned from virus in blood. Molecular assays targeting conserved genome regions of all EV types (5′UTR, 2 C, 3Dpol) were employed. As compared to direct assays of plasma or leukocytes, the EV detection rate was significantly enhanced by co-culture of leukocytes with cell lines prior to molecular and immunologic tests. Results of RT-PCR and sequencing were confirmed by staining cell cultures with a panel of EV-specific antibodies. Sequence and phylogenetic analysis showed that EVs of the C species (polioviruses) were associated with the post-polio syndrome, while members of the B species were found in type 1 diabetes and cardiomyopathy. The procedure may be used for investigating the possible association of different EVs with a variety of chronic neurologic, endocrine, and cardiac disorders.

## Introduction

Enteroviruses (EVs) are among the most frequent human pathogens and cause 10–15 million new infections per year in the USA^1^. Thus, over a lifetime, all people experience a number of different EV infections. The four EV species (A, B, C, D) contain 116 different EV types. EVs may cause either asymptomatic infection or acute illnesses ranging from diarrhea to paralysis and encephalitis^2^. EV infection in childhood provides lifelong protection against subsequent contact with homologous virus and may also contribute to preventing autoimmune disorders^3^.

Though most enterovirus infections appear to be self-resolving within a few weeks, prolonged fecal shedding of non-polio EVs and attenuated strains used in live poliovirus vaccine has been documented in normal children^4, 5^. Persistent EV infections are frequently encountered in immunodeficiency^6^, but long-term infection of immunocompetent hosts is still a matter of debate. Evidence is so far limited to certain neurologic^7, 8^, endocrine^9–11^, and cardiac disorders^12–15^. It is also known that vaccine-derived polioviruses may be isolated from sewage in places where no known immunodeficient persons live, suggesting that they can be shed by healthy persons chronically infected with the virus^16^.

Consequences of long-lasting infections are profoundly different from those of acute disease^17, 18^. Though EVs are cytolytic to their host cells^19^, some studies indicate that subclinical EV infection may be associated with lifelong disorders such as the post-polio syndrome (PPS)^20, 21^, myasthenia gravis^22^, autoimmune thyroiditis^23^, type 1 diabetes (T1D)^9, 10, 18^, chronic viral cardiomyopathy (CVC)^12–15^. The hypothesis that persistent EV infection may be linked to organ-specific autoimmunity and chronic disease is supported by recent results showing an incomplete humoral response to coxsackie B viruses in children who first developed insulin autoantibodies, then type 1 diabetes^24^ (T1D).

It is recognized that the spread of non-enveloped picornaviruses (e.g., HAV, CV-B3, EV-A71) within tissues is not always accompanied by cell death^25^ and that – in addition to free viral particles – these agents are also released from infected cells within membrane vesicles derived from the autophagy pathway^25^. The process may render viral particles resistant to antibody neutralization^26^.

Persistent EV infections have been studied in cultured cells of monkey, mouse, and human origin [e.g., the HEp2 line, neural and endothelial cells, astrocytes, neuroblastoma lines^27^]. Cultured cells undergoing persistent infection display peculiar features^27, 28^: a) cytopathic effect (CPE) is not evident, b) viral antigens are expressed in a small percentage of cells, c) virus is produced at low titers (≤10^3^ plaque-forming units/ml), d) cytokines and chemokines are secreted^29^. In persistently infected cultures, virus transmission may occur through the release of extracellular vesicles, formation of cell protrusions and intercellular bridges^8, 25, 26^. This exit mode may facilitate virus dissemination *in vivo* in the presence of a robust immune response^30^.

EVs undergo remarkable evolution linked to the high mutation rates common to all RNA viruses, intraclade recombination, deletions (5′UTR, 2 C regions)^8, 28, 31–33^. Thus, the genetic structure of current EV isolates is different from that of prototype strains that were obtained decades ago.

Public databases contain over 5,000 genome sequences of human EVs^1^. Members of an EV species share greater than 70 percent amino acid (AA) identity in the polyprotein, greater than 60 percent identity in P1, greater than 70 percent AA identity in the nonstructural proteins 2 C and 3CD^34^. Variability of AA sequences is highest in VP4 and 2 A and lowest in the 2 C, 3D, and 3 C regions^35^. Conserved sequences are also present in the internal ribosome entry site (IRES) located within the 5′ untranslated region (5′UTR) of the viral genome^2^.

The 5′UTR region comprises the targets of current commercial diagnostic assays for EV infections. These tests do not recognize the closely related human Parechoviruses (PeVs)^1^. Since low EV levels are produced in persistent infection^14, 28^ and the causative agents may carry genetic changes, commercial assays rarely give positive results in chronic disorders.

**Figure 1:**
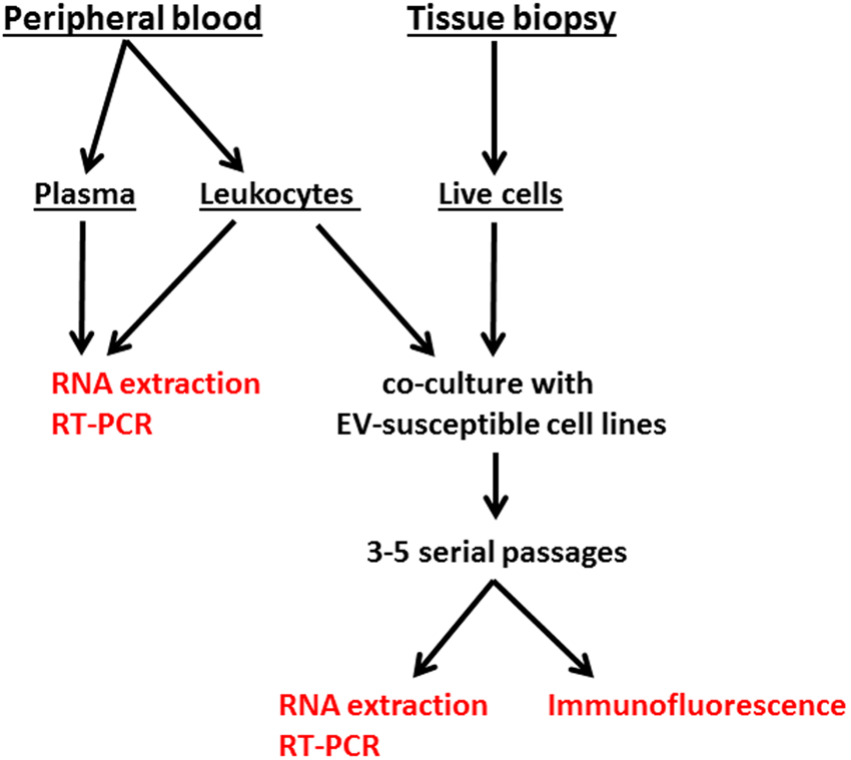
Representation of the integrated procedure used for detecting enteroviruses (EVs) in patients diagnosed with post-polio syndrome (PPS), type-1 diabetes mellitus (T1D), chronic viral cardiomyopathy (CVC). Venous peripheral blood or tissue biopsies were used as clinical samples. Plasma and blood leukocytes were tested directly by molecular assays (left side). Blood leukocytes and live cells obtained from biopsies were co-cultured with cell lines (right side). Cell cultures were serially passaged 3 to 5 times. RNA was then extracted from medium of cultured cells. Molecular assay were used to detect the viral genome. For each case, the expression of enteroviral antigens in cultured cells was evaluated by immunofluorescence with a panel of anti-EV antibodies.

To investigate the possible pathogenic role of long-term EV infection in different chronic disorders, we developed an integrated diagnostic approach based on virus growth in multiple cell lines^36^ followed by search for the viral genome and virus antigens (Fig. 1).

Conserved genome regions were interrogated with primer pairs specific to the 5′UTR, 2 C, and 3Dpol regions^37^. Expression of viral antigens in infected cultured cells was explored with a panel of EV-specific antibodies expected to confirm molecular results^38^.

As tissue-based approaches are centered on invasive surgical procedures which may be impossible in the majority of cases, information was mainly gleaned from virus circulating in blood. The procedure has been utilized to reveal EV infections associated with clinical cases of PPS, T1D, and CVC.

## Results

### Specificity and diagnostic range of molecular EV assays

The specificity of primer pairs targeting the 5′UTR, 2C and 3Dpol regions was evaluated versus EV types representative of the A, B, C, and D species. Table 1 shows the results of RT-PCR assays with EV types of the A, B, C, D species and with RNA viruses other than EVs (PeV-3, EMCV, HIV-1, HCV, measles, mumps, rubella virus). RNA extracted from uninfected cell lines and from a pool of blood donors’ leukocytes was used as negative control. With the exception of PeV-3 that was recognized by only one pair of 5′UTR primers^39^, tests for viruses other than EVs failed to produce amplicons. Likewise, no amplicons were obtained using RNA extracted from virus-negative controls. Primer pairs targeting the 5′UTR region showed the widest diagnostic range, while differing in the intensity of electrophoretic bands (Table 1). Primer pairs targeting the 2C or 3Dpol regions had similar sensitivity, but were more prone to amplify grouped EV types, i.e. members of the A, B, C, or D species. On the whole, results utilizing replication-competent EV strains closely reproduced the reactivity patterns predicted by bioinformatics analysis.

**Table 1:** Specificity of enterovirus primer pairs: RT-PCR amplification of enterovirus belonging to the A, B, C, D species and picornaviruses other than EVs^a^.

Genome region and primer pair	Virus type and enterovirus species													
	Negative Control^b^	CV-A2	CV-A16	EV-A71	CV-B3	CV-B4	Echo-16	CV-A24	PV-2	PV-3	EV-D68	EV-D94	PeV-3^c^	EMCV-D^d^
		A	A	A	B	B	B	C	C	C	D	D		
5′UTR-Nij^63^		+	+	+	+	+	+	+	+	+	+	+		
5′UTR-Tok^39^		+	+	+	+	+	+	+	+	+	+	+	+	
5′UTR-A		++	++	++	++	++	++		+	+		+		
5′UTR-B		+	++	++	+	++	++	+	+	+		++		
5′UTR-C		++	++	++	++	++	++	++	++	++	++	++		
5′UTR-D		+	+	+	+	+	+	+	++	+	+	++		
2C-1A			+	+	++	++	++		++	+	+	++		
2C-1B					+	++	+							
2C-2								+	++	+				
2C-3			++			+		++	+	+				
2C-4						+					++	++		
2C-5		++	+	++		+		+	+	+				
3Dpol-A		++	+	+										
3Dpol-B					+	+	++		+					
3Dpol-C								++	++	++				
3Dpol-D											++	++		
3Dpol PVs								+	++	+				

^a^Intensity of electrophoretic bands: blank, no band; +, medium; ++, strong. Data shown represent the results of PCR assays performed in duplicate.

^b^Supernatant of uninfected cell cultures.

^c^PeV-3, Parechovirus type-3.

^d^EMCV-D, Encephalomyocarditis virus a (D clone).

### Sensitivity of molecular EV assays and virus load in the supernatant of cell lines co-cultured with leukocytes of PPS and T1D patients

**Figure 2:**
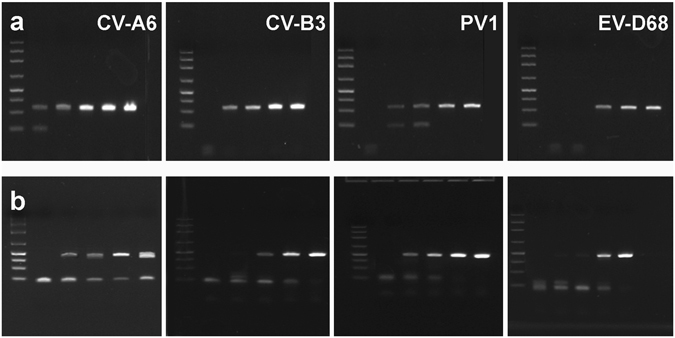
Results of end-point PCR of quantitated pU57 plasmids containing the 5′UTR region of CV-A6, CV-B3, PV-1, EV-D68 (representative enteroviruses of the A, B, C, and D species). Ten-fold dilutions of each plasmid were tested by end-point PCR with two different primer pairs: 5′UTR-Tok (panel a) and 5′UTR-C (panel b). For each gel, six lanes of cropped gels are shown (left to right): DNA size markers, PCR amplicons obtained with 1.6, 16, 160, 1,600, or 16,000 template molecules per reaction. Primer pairs were detecting ≤16 template molecules/reaction. Original, uncropped images are presented in the Supplementary Materials (Figures S1 and S2).

The sensitivity of novel molecular assays was tested by end-point PCR with ten-fold dilutions of pU57 DNA plasmids comprising the 5′UTR region of members of the A, B, C, and D species (CV-A6, CV-B3, PV-1, EV-D68). Representative results are shown in Fig. 2 [primer pairs 5′UTR-Tok (panel A) and 5′UTR-C (panel B)]. The assays could detect ≤16 DNA plasmid molecules/reaction, with sensitivity depending on the combination of primer pairs and different EV types. The sensitivity of amplification tests was confirmed by quantitative real time SYBR green assays utilizing the 5′UTR-Tok primer pair and the standard cycling profile reduced to 42 cycles. Results were confirmed by analysis of the dissociation profile (fluorescence readings collected at temperature increments from 55 °C to 95 °C) and are summarized in Table S4A. The median Ct value was 37.8 (range 37.0–38.2) for PPS cases, 35.7 for T1D cases (range 35.1–37.4). As shown in Table S4B, comparison with Ct values obtained using known copy numbers of pUC57-5UTR plasmids allowed to estimate the mean viral load of PPS samples at 177 genome copies/ml in cell culture medium (range 120–230/ml), and that of T1D samples at 470 genome copies/ml in cell culture medium (range 220–620/ml). Thus, the viral load in cell cultures exposed to samples of PPS and T1D patients is comparable to what documented in cardiac tissue of idiopathic cardiomyopathy [about 500 EV genome copies/μg of total extracted nucleic acids^15^]. The low virus levels indicate that replication-defective agents play a role in chronic EV infection.

In order to evaluate tests sensitivity under conditions identical to those adopted for clinical samples, biologically titrated EV strains representative of the A, B, C, D species (CV-A16, CV-B4, PV-2, EV-D94) were subjected to serial tenfold dilutions in complete medium. RNA was extracted from each dilution and utilized for reverse transcription. Primer pairs attained extreme sensitivity. Table 2 shows that molecular assays tended to remain positive with samples diluted up to 10^−9^

**Table 2:** End-point RT-PCR assays. Biologic titer of EV members of the A, B, C, D species and sensitivity of selected primer pairs^a^.

Enterovirus type (species)	Virus titer (CCID_50_/ml)	Dilution factor	Results of PCR assays using the indicated primer pairs^b^						
			5′UTR-Tok^39^	5′UTR-C	2C-1A	3Dpol-A	3Dpol-B	3Dpol-C	3Dpol-D
CV-A16 (A)	5.0 × 10^6^	10^−6^	+	+	+	+	nd^c^	nd	nd
		10^−7^	+	+	+	+	nd	nd	nd
		10^−8^	+	+		+	nd	nd	nd
		10^−9^		+			nd	nd	nd
CV-B4 (B)	6.5 × 10^6^	10^−6^	+	+	+	Nd	+	nd	nd
		10^−7^	+	+	+	Nd	+	nd	nd
		10^−8^	+	+	+	Nd	+	nd	nd
		10^−9^		+		Nd		nd	nd
PV-2 (C)	4.2 × 10^7^	10^−6^	+	+	+	Nd	nd	+	nd
		10^−7^	+	+	+	Nd	nd	+	nd
		10^−8^	+	+	+	Nd	nd	+	nd
		10^−9^	+	+	+	Nd	nd	+	nd
EV-D94 (D)	1.8 × 10^7^	10^−6^	+	+	+	Nd	nd	nd	+
		10^−7^	+	+	+	Nd	nd	nd	+
		10^−8^	+	+	+	Nd	nd	nd	+
		10^−9^	+	+	+	Nd	nd	nd	

^a^Virus titer is expressed as cell culture infectious doses_50_/ml (CCID_50_). Total RNA was extracted from appropriate virus dilutions in cell culture medium and reverse transcripted. PCR assays were then run as reported in Methods.

^b^Results of end-point RT-PCR. Positivity assigned to visible bands of the expected size in 2.2% agarose gels.

^c^nd, not done.

. Thus, at least for the investigated EV types, the sensitivity of PCR assays targeting the 5′UTR, 2 C, and 3Dpol regions exceeded by 10 to 100-fold that of biologic virus titration.

Using the same virus strains and plasmids reported above, the sensitivity of eight different commercial real time EV assays intended “for research use only” (Table S1) ranged from 10^3^ to 10^4^ copy numbers/ml (data not shown).

While it is difficult to explain why persisting EVs fail to reach normal virus titers in susceptible cell lines, the exceedingly low amounts of virus found in cell culture media appears to justify the lack of CPE perceivable by microscopy.

### Replication-defective EVs maintain infectivity upon filtration through 100 nm membranes

Cell-free supernatant of cells co-cultured with leukocytes of PPS, T1D, and CVC cases could transmit infection to uninfected cell. As proven by RT-PCR and indirect fluorescence with panenterovirus MAbs, culture-to-culture transmission of infection resisted filtration through 220 and 100 nm membranes (data not shown).

In an attempt to determine the replicative capacity of persisting EV isolates under different conditions, AV3 and RD cell lines were infected with virus isolates obtained from PPS, T1D, and CVC isolates. Cells were incubated at 31 °C or 36 °C. Using RT-PCR assays and indirect immunofluorescence, no differences were detected in virus levels produced at different temperatures (3, 6, 9 days post-infection; data not shown). Thus, persisting EV isolates could not be designed as temperature-sensitive.

### RT-PCR assays: enterovirus detection directly in plasma or blood leukocytes vs. virus retrieval in medium of infected cell-cultures

Samples of blood donors and patients diagnosed with either PPS, T1D, or CVC were investigated. Demographic data of the investigated subjects (including control blood donors) are summarized in Table 3

**Table 3:** Demographic data of control blood donors and patients diagnosed either with Post-Polio Syndrome (PPS), Type 1 Diabetes (T1D), or Chronic Viral Cardiomyopathy (CVC).

Investigated subjects	Blood donors	Clinical cases		
		PPS	T1D	CVC
No. cases	15	17	14	10
Median age, years (range)	33.0 (18–52)	65.5 (54–79)	8.3 (3.8–16.3)	23.5 (16–51)
Time from clinical onset to sampling (range)		22–78 years	1–16 days	2–23 months

. The time from clinical diagnosis to collection of the first sample varied among different groups. With regard to PPS cases, since poliomyelitis had been diagnosed within the first two years of life in our patients, collection of samples occurred 22–78 years post-diagnosis. T1D samples were instead collected at the time of clinical onset (range 1–16 days). CVC samples were obtained 2–23 months post-diagnosis.

In all cases, plasma and peripheral blood leukocytes were tested directly by RT-PCR for the presence of EV genomes. In addition, separated blood leukocytes were co-cultured with cell lines for 3–6 serial passages. The EV positivity rate achieved with direct tests was compared to that attained with samples which had been co-cultured with cell lines^36^ before molecular tests. Results are summarized in Table 4. Using direct tests, none of the controls was EV-positive and only 1/15 controls became positive upon co-culture of leukocytes with cell lines. Among patients, the EV detection rate in plasma was 8/41 vs. 16/41 in leukocytes (P = 0.082). The comparison suggests that virus is harbored in blood leukocytes. As seen in Table 4, when leukocytes were co-cultured with cell lines before molecular tests, the positivity rate reached 32/41 cases. Thus, using a pre-culture step before RT-PCR assays significantly boosted virus detection as compared to direct assays of either plasma (p < 0.0001) or leukocytes (p < 0.0007).

**Table 4:** Comparison of virus positivity rates in plasma and in blood leukocytes versus blood leukocytes that had been co-cultured with cell lines before molecular assays^a^.

Sample	No. enterovirus-positive/total (%)					
	Blood donors	Clinical cases			Total	P^b^
		PPS	T1D	CVC		
Plasma^c^	0/15	2/17	4/14	2/10	8/41 (19.5%)	
Blood leukocytes^c^	0/15	6/17	6/12	4/10	16/41 (39.0%)	0.082^e^
Blood leukocytes co-cultured with cell lines before molecular assays^d^	1/15	14/17	11/14	7/10	32/41 (78.1%)	<0.0001^f^ <0.0007^g^

^a^EV-specific RT-PCR assays performed with primer pairs 5′UTR-A, -B, -C, -D as in the Methods section. Positivity assigned to visible bands of the expected size in agarose gels.

^b^Fisher’s exact test, two-sided.

^c^Plasma and blood leukocytes: direct RNA extraction, then RT-PCR.

^d^Blood leukocytes co-cultured with cell lines for 3–6 passages. RNA was extracted from cell culture medium, then RT-PCR.

^e^P value: blood leukocytes vs. plasma.

^f^P value: co-culture method vs. plasma.

^g^P value: co-culture method vs. blood leukocytes.

### Sequences of EV amplicons obtained from cases of persistent infection

**Figure 3:**
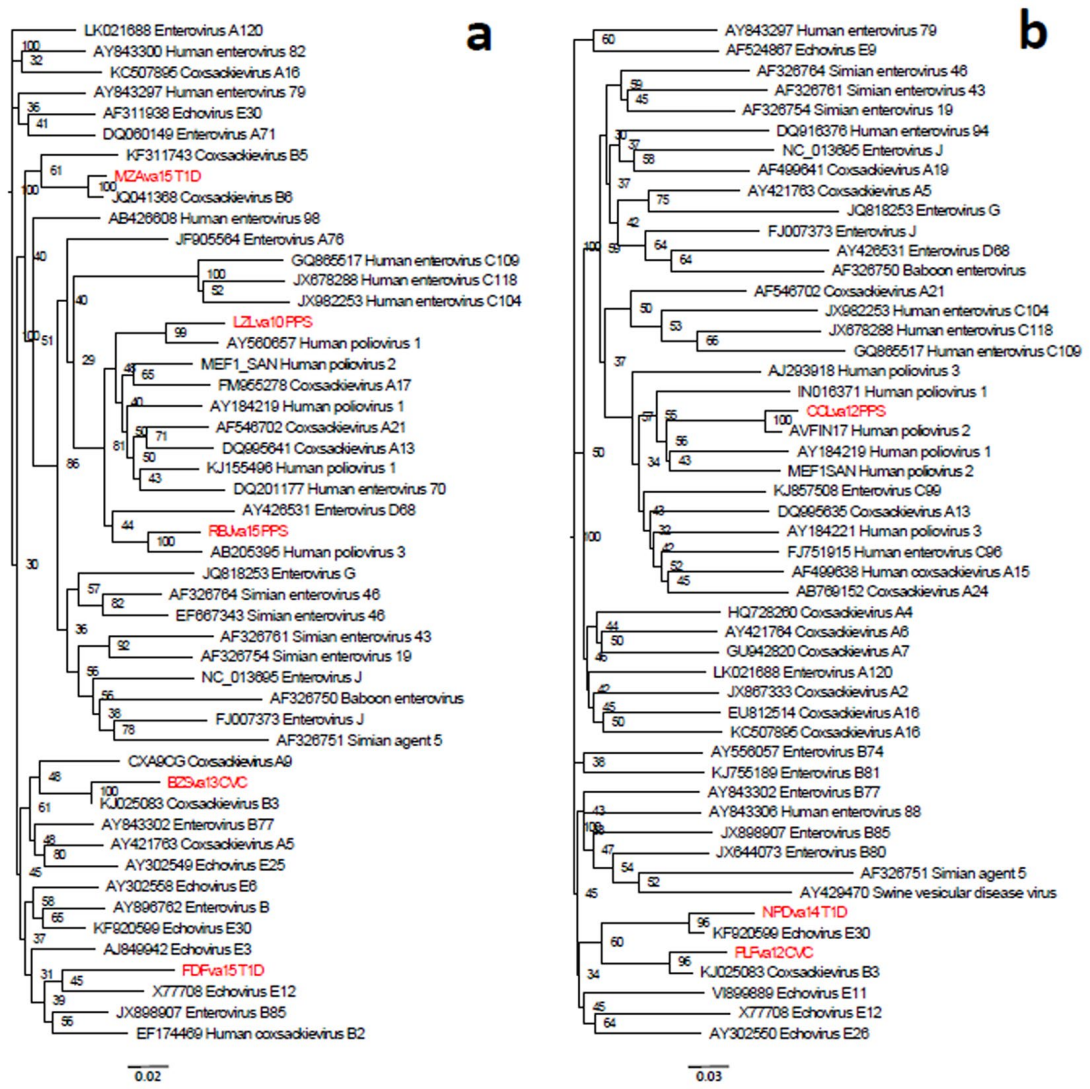
Phylogenetic analysis of nucleotide sequences of a part of the 5′-UTR region (nucleotides 345–607, panel a) and a region covering the distal part of 5′-UTR, VP4 and the proximal part of VP2 (nucleotides 580–1170, panel b) determined for PCR amplicons. Sequences determined for PPS, T1D, and CVC cases are shown in red. Homologous regions from a selected set of 50 published reference enterovirus sequences were used to determine their relatedness using alignment-free k-mer mismatch method. Phylogenetic trees were reconstructed from the distance matrix using neighbor-joining method. The quality of each internal branch of the tree was assessed as a percentage of all possible 4-node subsets that confirmed the local topology of the tree.

Direct sequencing of EV amplicons obtained from PPS, T1D, or CVC cases was not easy, possibly due to the very low virus levels, to the presence of “virus quasispecies”^31, 40^, and to the contamination of cultured virus with host cell nucleic acids^41^. In fact, chromatograms frequently contained virus sequences mixed with additional traces often attributable to human genes. Some sequences, however, gave hints to different EVs (Table 5). In selected patients, virus detection remained positive at different times confirming the persisting/chronic nature of infection, and extremely similar sequences could be obtained in cases that were analyzed at different times. Phylogenetic trees based on a comparison of PCR amplicon sequences (partial 5′UTR and 5′UTR-VP2 regions) with homologous regions of nucleotide sequences of a representative set of EVs showed that amplicons of PPS cases clustered with polioviruses, whereas amplicons of T1D and CVC cases clustered with non-polio EVs of the B species (Fig. 3).

**Table 5:** Sequence of enterovirus amplicons obtained from cases of post-polio syndrome (PPS), type 1 diabetes (T1D), and chronic viral cardiomyopathy (CVC).

Diagnosis	Case	Genome Region	Amplicon Sequence	Closest match in BLAST			
				Accession No.	Description	Identities, gaps	EV species
PPS	RBJ/va15	5′UTR	GGCTAATCCTAACGATGGAGCACGCAGCTGCAACCCTGCAGCCAACCTGTCTTAACGCGCAAGTCCGTGGCGGAACCGACTACTTTGGGTGTCCGTGTTTCCTTTTATTCTTGAATGGCTGCTTATGTGACAATCACAATCA	KX162706.1	Human poliovirus 2 strain NIE0611579	131/137, 1	C
		5′UTR	TGGTGTTGGTGTGAAGAGCATATTGAGCTACATGAGAGTCCTCCGGCCCCTGAATGCGGCTAATCCTAACGATGGAGCACGCAGCTGCAACCCTGCAGCCAACCTGTCTTAACGCGCAAGTCCGTGGCGGAACCGACTACTTTGGGTGTCCGTGTTTCCTTTTATTCTTGAATGGCTGCTTATGTGACAATCACAATCA	KU763188	Human poliovirus 3 isolate 45507, Sabin-derived	180/187, 1	C
	LZL/va10	5′UTR	TCATGAGATCCTCCGGCCCCTGATGCGGCTAATCCTAACCATGGAGCAGGTAATCGCAAACCCAGCGGTCAGCCTGTCGTAACGCGTAAGTCTGTGGCGGAACCGACTACTTTGGGTGTCCGTGTTTCCTTTTATTTTTATGGGGGTGGTTAAGGGGGCCATCCAGA	KP793687.1	Human poliovirus 1 strain Brunenders	151/163, 4	C
	CCL/va12	5′UTR	GCTGCTTTATGGTGACAATCAGAGATTGTTATCATAAAGCGATTTGGATTGGCCATCCGGTGTGTGTTGCATCAAATACGTTAATACTTGTTTAAACTATTGTATTAATTTTACCCTTCTCTTAATCAATCACTCATAAACACTACGAGGATTGAATTACAGT	DQ890387.1	Human poliovirus 2 strain USA9810768, Sabin-derived from chronic immunodefficient patient	132/163, 5	C
		5′UTR-VP2	GCTGCTTTATGGTGACAATCAGAGATTGTTATCATAAAGCGATTTGGATTGGCCATCCGGTGTGTGTTGCATCAAATACGTTAATACTTGTTTAAACTATTGTATTAATTTTACCCTTCTCTTAATCAATCACTCATAAACACTACGAGGATTGAATTACAGTACTACAATGGGAGCCCAAGTGTCGACACAGAAAGTCGGAGCTCACGAAAATTCAAATAGAGCCTATGGCGGGTCCACCATCAATTACACTACAATCAATTATTACAGAGATTCTGCAAGCAGTGCTGCGAGCAAGCAAGATTTTGCTCAAGACCCATCCAAGTTCACTGAGCCCATCAAGGATGTCCTCATAAAGACCGCACCCATGCTGAACTCCCCGAACATTGAGGCGTGTGGTTACAGTGATAGAGTAATGCAATTAACTCTGGGTAACTCAACAATCACCACTCAAGAGGCGGCCAACTCCGTTGTTGCTTACGGCAGATGGCCTGAATACATCAGAGATTCTGAGGCAAATCCCGTGGACCAACCAACCGAACCCGACGTGGCTGCGGTGCAGGTTTTACACGTTGGACGCCCAGAGCTTTGAATAAAGCTGGGAT			495/579, 6	C
T1D	MZA/va15	5′UTR	GTTGGCGGCCAGCCCACTGGGGCAACCCATGGGACGCTTCAATACTGACATGGTGCGAAGAGTCTATTGAGCTAATTGGTAGTCCTCCGGCCCCTGAATGCGGCTAATCCTAACTGCGGAGCAGGCACTCGCAGACCAGCGAGCAGCTTGTCGTAATGGGCAACTCCGCAGCGGAACCGACTACTTTGGGTGTCCGTGTTTCCTATTTCCTTTATATTGGCTGCTTATGGTGACATATAAGGAAA	AF114384.1	Coxsackievirus B6 strain Schmitt	231/235, 1	B
		5′UTR	TGGCTGCGTTGGCGGCCAGCCCACTGGGGCAACCCATGGGACGCTTCAATACTGACATGGTGCGAAGAGTCTATTGAGCTAATTGGTAGTCCTCCGGCCCCTGAATGCGGCTAATCCTAACTGCGGAGCAGGCACTCGCAGACCAGCGAGCAGCTTGTCGTAATGGGCAACTCCGCAGCGGAACCGACTACTTTGGGTGTCCGTGTTTCCTATTTCCTTTATATTGGCTGCTTATGGTGACA			238/242, 1	B
	FDF/va15	5′UTR	GTCCTCCGGCCCCTGAATGCGGCTAATCCTAACTGTGGAGCAGGATAGCCTGACAGACACCGCTGGCCCAGCCCTGTCGTAACGGGCAAGCTCTGTCGAGCGGAACCGACTACTTTGGTGACCGTGTTTCA	JX898908.1	Coxsackievirus B1 strain Groningen/2011	112/131, 11	B
	NPD/va14	5′UTR	ATGTTGCCATATAGCGATTGGCTTGGCCTCCGGTGTCCAATAAAGCGATCATTTATTTGTTTGTTGGGTTCGTACCCTTGAATTACAAAGCAATTGTAACGCTTACCTAACTGCTGTGCCACTGAATTCAGATCTCCCGGGCCA	AM237001.1	Human echovirus 30 isolate CF2249-01	95/105, 1	B
		5′UTR-VP2	CTACTGCGGACAGAACCTACACGCCAGTGGGCAGTCTGTCGTAACGGGCAACTCCGCAGCGGAACCGACTACTTTGGGTGTCCGTGTTTCCTTTTTCTTTATACTGGTTGCTTATGGTGACAATTGAGAGATTGTTGCCATATAGCTATTGGCTTGGCCATCCGGTGTCCAATAAAGCGATCATTTATTTGTTTGTTGGGTTCGTACCCTTGAATTACAAAGCAATTGTAACGCTTAAATATATTATAGACCTCAACACAGCAAAATGGGGGCCCAAGTTTCAACACAAAAAACTGGAGCTCATGAGACCGGCTTGAGTGCCAGTGGAAACTCCATTATACATTACACAAATATTAATTACTACAAGGACTCTGCTTCTAACTCATTGAGCCGGCAAGACTTTACCCAAGATCCCAGTAAATTCACAGAACCGGTGAAGGATGTGATGATTAAGACCTTGCCCGCTTTAAATTCACCCACTGTAGAAGAATGCGGCTTCAGTGACCGAGTGCGGTCGATCACCCTGGGGAATTCTACGATCACCACACAGGAGTGTGCTAACGTGGTTGTGGGATAC		Human echovirus 30 isolate CF1570-02	550/578, 3	B
CVC	BZS/va13	5′UTR	GCCTCCGGCTCCTTAAGGTGACTAATCCCTAACTGCGGAGCACACACCCTCAAGCCAGAGGGCAGTGTGTCGTAACGGGCAACTCTGCAGCGGAACCGACTACTTTGGGTGTCCGTGTTTCATTTTATTCCTATACTGGCTGCTTATGGTGACAATCACCC	KF986401.1	Human coxsackievirus B3 strain MCCV1S	149/155, 1	B
	PLF/va12	5′UTR	TGGTTGGTGGGCCTCCGGCTCCTTAAGGTGACTAATCCCTAACTGCGGAGCACACACCCTCAAGCCAGAGGGCAGTGTGTCGTAACGGGCAACTCTGCAGCGGAACCGACTACTTTGGGTGTCCGTGTTTCATTTTATTCCTATACTGGCTGCTTATGGTGACAATTTGAGAGATTGTTCCATATAGCTATTGGATTGGCCACTACTTGATGCCCCGGGAGCTATTATATATCTCTTTGTTGGGTTTATACCACTTAGCTTGAAAGAGGTTAAAACATTACAATTCATTGTTAAGTTGAATACAGCAAAATGGGAGCTCAAGTATCAACGCAAAAGACTGG	KJ025083.1	Human coxsackievirus B3 strain MKP	319/340, 5	B
		5′UTR-VP4	TGGTTGGTGGGCCTCCGGCTCCTTAAGGTGACTAATCCCTAACTGCGGAGCACACACCCTCAAGCCAGAGGGCAGTGTGTCGTAACGGGCAACTCTGCAGCGGAACCGACTACTTTGGGTGTCCGTGTTTCATTTTATTCCTATACTGGCTGCTTATGGTGACAATTTGAGAGATTGTTCCATATAGCTATTGGATTGGCCACTACTTGATGCCCCGGGAGCTATTATATATCTCTTTGTTGGGTTTATACCACTTAGCTTGAAAGAGGTTAAAACATTACAATTCATTGTTAAGTTGAATACAGCAAAATGGGAGCTCAAGTATCAACGCAAAAGACTGGGGCACATGAGACCGGGCTGAATGCTAGCGGCAATTCCATCATTCACTACACAAATATTAATTATTACAAGGATTAGCCACATT			392/413, 5	B
	LZS/va09	3Dpol	TCTTCTATCCCTGGCCTATAAACTCTTCCCCGTTGGCGCACATTGGTAAGGGTTACGGATTGATCATGACACCAGCTGATAAAGGAGAGTGTTTCAATGAAGTCACCTGGACAAACGTCACCTTCCTGAAAAGGTATTTCAGAGCAGATGAGCAATATCCATTCCTAGTACATCCAGTCATGCCCATGAAAGATATACATGTTTCTATTCGATGGACTTATGACCCAC	U05852.1	Echovirus 6 non-lytic persistent strain	189/227, 1	B
		5′UTR	ACCAATTACTTCGAGATTCCTAGTATCACCATCAAGGTTGGGCAGTGTTACCCTCCGCACAACTTCAGTGTAGATCAGGCCGATGAAGCACCGCACTCCGTACGGGCGACCGTGGCGGTGCCTGCGCTGGCGGCTAGCCCATATCCCAACCCATGGGCCGCTTCAAAACTGACTTGGTCTGAACTGTCTATTGAGCTAATTGGTAGTTATCCGGCCCCTGAATGGGCCTAATCCTAACTGCGCCGCAGATACCTACATGCCAGTCTGCGGAGTCTGTCGTAACGGGCAACTCCGCGGCGGAATCGACTACTTTGGGTGTGGGTGTTTCCTGTTATTTTATTCTGGGTTGTTATTGGGACCATTGAGAGATTGTTATCA	AB705311.1	Echovirus 6 strain Hokkaido/19724/2011	330/379, 3	B

**Figure 4:**
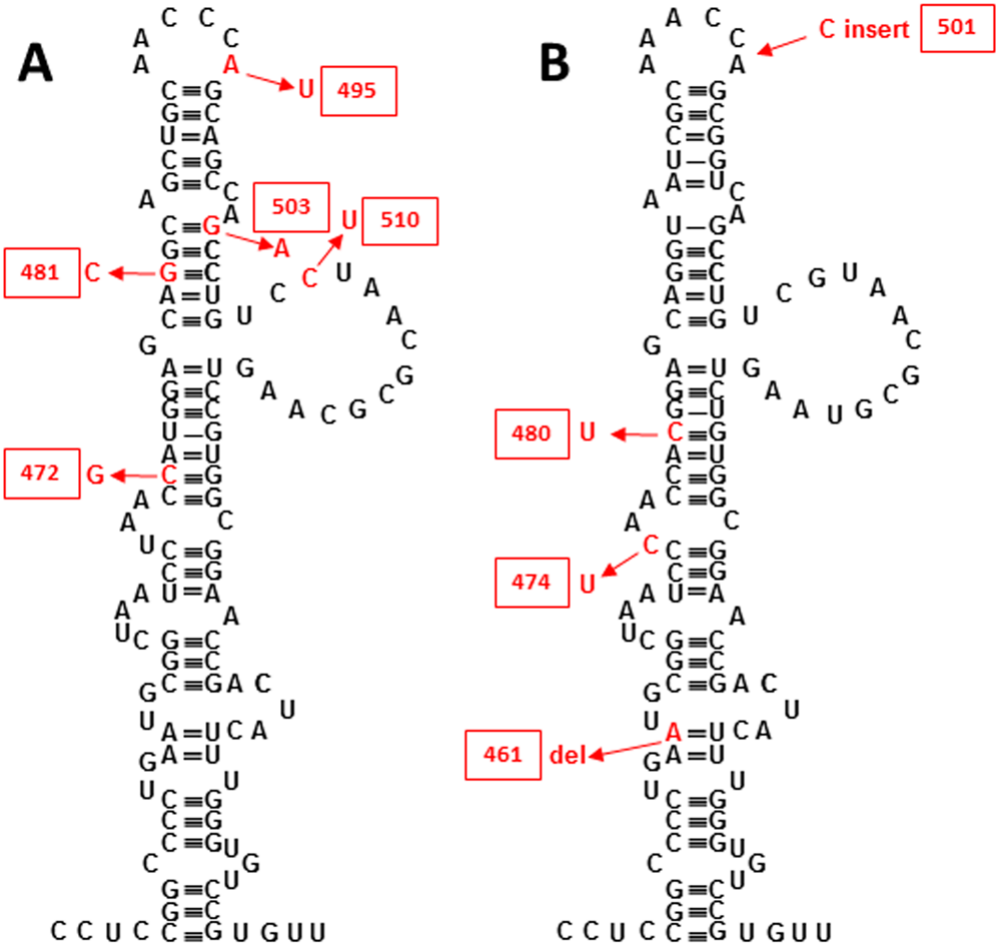
Secondary structure of the IRES element domain V from PPS cases RBJ/va15 (**A**) and LZL/va10 (**B**). Hairpin structures are presented in black for viruses that are most closely related to the PCR product sequences from respective cases. Mutations in the sequences from PPS cases are shown in red. In most cases these mutations disrupt the secondary structure of domain V, suggesting that the mutated agents have reduced fitness.

Sequences of PPS amplicons closely matched poliovirus sequences. As shown in Fig. 4, partial 5′-UTR sequences from PPS cases represent domain V in which a major neurovirulence determinant is located. Sequence from cases RBJ/va15 and LZL/va10 were related to vaccine-derived strains of poliovirus type 3 and type 2, respectively. Mutations disrupted the secondary structure of domain V which is related with neurovirulence and fitness of poliovirus. The sequence from case CCL/va12 represents the end of 5′-UTR, the entire VP4, and the beginning of VP2 genes. The amino acid sequence of VP4 was identical to an evolved vaccine-derived type 2 poliovirus isolated from the environment, and the overall similarity of the 605-nucleotide segment was 94%.

With regard to T1D and CVC cases, the partial sequence of 5′-UTR-VP4-VP2 genes of the T1D case NPD/va14 was 97% similar to Echovirus 30, with 99% similarity of the amino acid sequence. The amino acid sequence derived from a 3Dpol PCR amplicon of CVC case LZS/va09 was 100% identical to amino acids 355–400 of the 3D polymerase of a number of EV-B viruses (Coxsackie B1, B2, B3, B4, B5, B6, A9, Echo-11). This region codes for the outer surface of the “thumb” domain which is outside of the catalytic site. While the closest match at the nucleotide level was coxsackievirus B2 (91% similarity), the highly conserved nature of this region makes it difficult to determine the serotype identity of the agent associated with the case. Therefore, T1D and CVC cases were associated with non-polio EVs (especially of the B species; Table 5).

Unfortunately, sequencing of the VP1 capsid protein gene was unsuccessful, though multiple methods effective for replication-competent EVs were utilized^39, 42–44^.

### Cytopathic effect and immunofluorescence with antiviral antibodies

**Figure 5:**
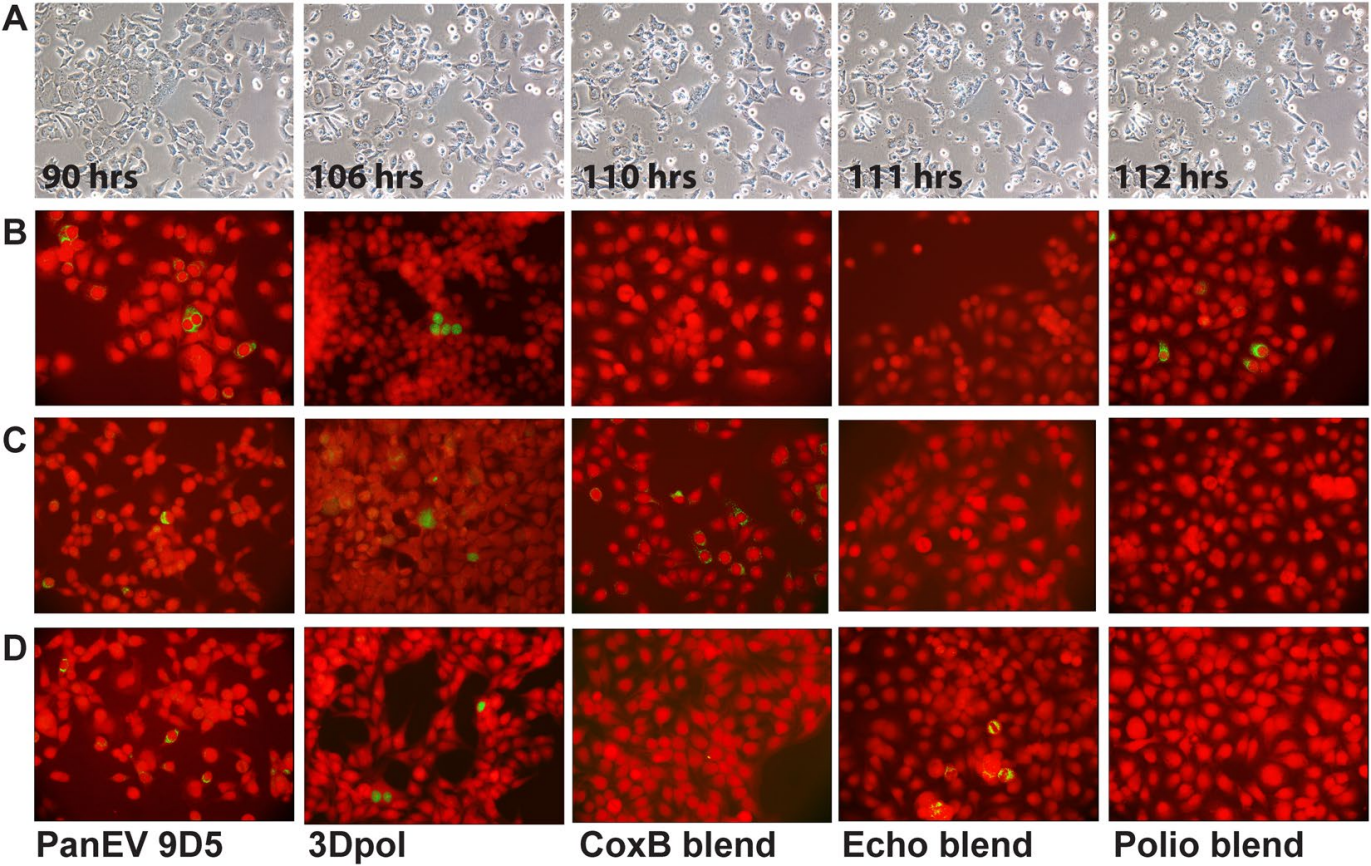
Slow development of morphologic changes evidenced by time-lapse microscopy in cell cultures infected with a viral isolate obtained from a T1D case and serially passaged 4 times (Row **A**). Incubation times are indicated in hours. Pictures were taken at 30 minutes intervals. Death of scattered cells was first observed after >100 hr incubation. Ballooning of scattered cells started appearing at 110 hrs. Then, in a couple of hours, cells were bursting with the release of multiple small vesicles (111 and 112 hrs). Original magnification 10x. Indirect immunofluorescence of human AV3 cell monolayers that had been co-cultured with leukocytes of patients diagnosed with PPS, T1D, or CVC and serially passaged 4–6 times. A panel of MAbs against EVs was used (green staining); Evans blue counterstain (red). Original magnification 20x. (Row **B**) cell monolayers exposed to leukocytes of a PPS case. Panenterovirus MAb 9D5 directed to VP1 and MAb 3D-05 directed to the 3D viral RNA polymerase produced cytoplasmic or nuclear staining in 1–2 percent of cells. MAb blends against coxsackieviruses B or echoviruses failed to detect virus antigens, whereas a blend of MAbs to poliovirus-1, -2, -3 produced clear cytoplasmic fluorescence. The PPS case was associated with an EV belonging to the poliovirus group (**C** species). (Row **C**) cell monolayers exposed to leukocytes of a T1D case. MAbs 9D5 and 3D-05 produced cytoplasmic staining in 1–2 percent of cells. The MAb blend to coxsackieviruses B produced granular cytoplasmic fluorescence; no staining was obtained with MAbs to echoviruses or polioviruses. The T1D case was associated with a non-polio EV, possibly belonging to the Coxsackie B group (B species). (Row **D**) cell monolayers exposed to leukocytes of a CVC case. MAbs 9D5 and 3D-05 produced cytoplasmic or nuclear staining in 1–2 percent of cells. The MAb blend to coxsackieviruses B did not detect virus antigens, whereas a MAb blend to echoviruses produced granular cytoplasmic fluorescence. No staining was obtained with poliovirus MAbs. This case was associated with a non-polio EV possibly belonging to the Echovirus group (**B** species).

While cytopathic effect could not be perceived by standard microscopy, prolonged time-lapse microscopy of cell lines exposed to leukocytes of PPS, T1D, or CVC cases allowed to substantiate the lysis of occasional cell clusters coupled with cell ballooning and the release of small vesicles spreading out of dead cells. Figure 5 (row A) shows the development of CPE in AV3 cell cultures that had been exposed to leukocytes of a T1D case and serially passaged six times. Notably, CPE manifested itself after more than four days observation. Cell death became evident in just a couple of hours (cell ballooning and release of minute vesicles). Phenomena of this type could not be observed in uninfected cell lines or in cell lines exposed to leukocytes of healthy blood donors.

Interesting findings emerged when cell lines exposed to leukocytes of patients with chronic disorders and subjected to serial passages were stained with MAbs directed to different EV groups and to the enteroviral 3D RNA-polymerase. At appropriate dilutions, EV-specific antibodies produced no staining in uninfected cell monolayers (data not shown).

In AV3 and RD cells co-cultured with blood leukocytes of PPS, T1D, or CVC cases, 0.1 to 2 percent of cells were expressing EV antigens. In particular (Fig. 5, row B), AV3 cells from a PPS case (RBJ/va15) showed cytoplasmic staining with the panenterovirus MAb 9D5 that targets VP1 and nuclear staining with the 3Dpol-05 MAb that targets the viral 3D RNA polymerase. The finding indicates that not only viral capsid proteins are expressed in persistently infected cell cultures, but also the RNA polymerase, an enzyme essential for virus replication. In contrast, cell monolayers were not stained by MAbs against coxsackie B viruses and echoviruses. A blend of MAbs to polioviruses (type 1, 2, -3) produced clear cytoplasmic fluorescence. The finding was confirmed with a humanized chimpanzee MAb capable of neutralizing poliovirus type-1 and -2^45^ and with rabbit anti-polio antibodies (data not shown). Thus, combined with results viral sequences that point to the C species EV for all PPS cases (Table 5), immunofluorescence indicates that cells exposed to leukocytes of case RB1/va15 were carrying a poorly-replicating variant of poliovirus. This novel finding is of obvious pathogenic and epidemiologic interest^21^.

Similar results were obtained with cell lines exposed to samples of patients diagnosed with T1D (Fig. 5, row C). In AV3 cells co-cultured with leukocytes of a T1D case (MAZ/va15), granular cytoplasmic fluorescence was seen in about 1 percent cultured cells incubated with either MAb 9D5 or 3Dpol-05. A blend of MAbs to CV-Bs gave cytoplasmic fluorescence, whereas no staining was seen with MAbs to echoviruses or polioviruses. Lack of reactivity with poliovirus MAbs was confirmed using rabbit anti-polio sera (data not shown). This T1D case was associated with infection by a non-polio EV possibly belonging to the B species.

For CVC case LZS/va09, MAbs 9D5 and 3Dpol-05 gave cytoplasmic or nuclear fluorescence. MAbs to coxsackie B viruses and polioviruses gave no staining, echovirus MAbs gave intense cytoplasmic positivity (Fig. 5, row D). Thus, presumably, this CVC case was associated with an echoviral infection.

In summary, immunofluorescent microscopy confirmed that EV belonging to different taxonomic groups may be linked to different chronic disorders of suspect viral etiology. In particular, polioviruses (C species EVs) are associated with PPS. The results corroborate the view that samples of patients with different chronic disorders are carrying EVs of different groups and that these persisting agents can endure serial passage *in vitro*.

### Sensitivity of immunofluorescence versus molecular assays

Results of indirect immunofluorescence substantiated the specificity of EV signals obtained through gene amplification using multiple primer pairs and different techniques. In all cases, the percentage of cells stained by EV antibodies and the intensity of fluorescent signals were directly proportional to the results of RT-PCR assays. Cultures that were negative by immunofluorescence consistently gave negative results by gene amplification. Thus, the sensitivity of indirect immunofluorescence was comparable to that of molecular methods.

## Discussion

The proposed diagnostic approach that exploits virus enrichment in cell culture before gene amplification and immunostaining can be used in a variety of clinical conditions where persistent EV infections are suspected. This procedure will stimulate research on chronic pathologies possibly associated with these infectious agents.

Our results do not clarify how poorly-replicating EV variants can persist in a minority of cultured cells without producing manifest CPE. However, CPE could be detected in occasional cells using prolonged observation. By culturing infected cells at reduced temperature (31 °C) we were unable to enhance virus replication. Similar results have been reported in cultured human astrocytes and in other models of persistent EV infection^8, 27^, thus indicating no role for temperature-sensitive mutants. An important result is that infection by persistent EV strains could be passed to uninfected cells upon filtration through 100 nm membranes. This is consistent with the 27–30 nm size of these agents and indicates their possible transmission among individuals.

A remarkable result of the study is the finding, in PPS cases, of virus sequences related to Sabin strains of poliovirus. A 605 nucleotide-long sequence detected in one patient was 94% similar to a highly evolved vaccine-derived poliovirus type 2. This suggests that PPS case CCL/va12 may be related to prior infection with vaccine poliovirus. Similarly, nucleotide and amino acid sequences of VP4 and VP2 proteins of T1D case NPD/va14 appear to be strongly related to echovirus-30. Amino acid sequences of the PCR amplicon from 3Dpol region were identical to the sequence of the outer surface of the “thumb” domain of viral polymerase of a number of EV-B viruses. At the nucleotide level, the closest match was coxsackievirus B2, with similarity at the level of within-serotype differences (91%).

Virologists have provided evidence for naturally-occurring enteroviral isolates characterized by 5′UTR deletions in human myocardium^13, 15, 46^ as well as in mouse pancreas and myocardium^32, 46^. The 5′UTR region of EVs includes the internal ribosomal entry site (IRES) consisting of five domains. Domain V is involved in virulence and in the initiation of translation^47^. Disruption of the secondary structure of domain V leads to virus attenuation and reduced fitness. Therefore domain V mutations found in sequences from PPS cases may contribute to the persisting nature of infection along with mutations in other parts of the genome.

Our results are subject to at least two limitations. First, identification of EV isolates could not be obtained via sequencing the capsid-coding regions (mainly the VP1 gene) according to published procedures^39, 42–44^, nor using poliovirus-specific PV1 primers. In prospect, identification of defective EV types may be achieved using sequencing tools of enhanced sensitivity^48^, or via sequencing cDNA fragments subjected to preliminary enrichment with primer sets covering the entire genome of EV groups/species^43^. In addition, novel cell culture methods allowing serial passage of persistent EVs at sufficiently high titers may also favor sequencing.

Nonetheless, using current technologies, phylogenetic trees of partial sequences indicate that amplicons of PPS cases are clustering with polioviruses, whereas amplicons of T1D and CVC cases are associated with non-polio EVs of the B species (Fig. 3).

Second, our studies did not permit identifying the EV-carrying cell types. Studies of cells other than leukocytes could have given pathogenesis-relevant information. For instance, some duodenal biopsies of PPS patients were EV-positive, indicating that gut cells may harbor EVs as previously reported for T1D patients^49^. Thus, it will be important to search for viruses in neural cells of PPS cases, pancreatic cells in diabetes, cardiac cells in CVC.

While neuropathology studies of PPS using either EV antibodies or modern genomics have not yet been reported^50^, studies with EV antibodies have been performed in myocardial tissue of patients undergoing heart surgery and in pancreatic tissue of diabetics. EV capsid antigens are expressed in cardiomyocytes of subjects with chronic cardiomyopathy^14, 46, 51, 52^, as well as in pancreatic beta cells^53–55^ and lymphoid cells of diabetic patients^29, 56^. Notably, Norwegian investigators succeeded in detecting EV infection in pancreata of type 1 diabetics at the clinical onset^9, 57^. Taken together, the results of the above studies suggest that EV persistence and organ damage are causally related.

The high sensitivity of the reported procedure makes it suitable to identifying the types of EV-carrying leukocytes in blood or lymphoid organs. Methods for separating lymphoid cell subpopulations are commonplace and can be exploited to learn whether monocyte/dendritic cells do represent EV carriers in neurologic and cardiac disorders, as already proposed for T1D^56, 58^ and myasthenia gravis^22^.

In our case, the repeated detection of highly similar EV sequences at different times post-diagnosis supports the case of prolonged subclinical infection in PPS, T1D, and CVC. However, distinguishing between a causative or a cofactor role for these infections requires further research and, possibly, interventional studies^52^.

The proposed procedure may help identifying viral factors that trigger or cause disorders with genetic and immunologic components, such as T1D^10, 18^ or the nephrotic syndrome in childhood^59^. However, culture methods have largely been discontinued in most laboratories and direct molecular detection is now the preferred method for diagnosis^1^.

Thus, some obstacles are remaining before detection and characterization of poorly-replicating EVs may support the clinical assessment of chronic disorders. This is especially true for complex procedures that require virus growth *in vitro* supplemented with genomic and immunologic assays. Nonetheless, the association of enteroviral pathogens with human diseases is gradually becoming unequivocal and the medical community is showing a rising interest in antipicornaviral drugs^60, 61^ and vaccines^62^.

## Methods

Consumables, cell lines, culture media, chemicals, molecular biology reagents, antiviral antibodies, commercial molecular assays for EVs and polioviruses: Table S1.

### Clinical samples

Cases of PPS, T1D, or CVC were diagnosed and followed-up at the Clinical Center of the University of Insubria (Varese, Italy). The observational study was approved by the Ethics Committee of Ospedale di Circolo and Fondazione Macchi (Varese, Italy) and was performed in accordance with The Declaration of Helsinki and local regulatory laws. Informed consent was obtained from all subjects. Blood was collected in K_2_EDTA Vacutainer tubes. Within 2 hrs, mononuclear cells and granulocytes were separated by centrifugation on Ficoll-Hypaque (density 1.077 and 1.119 g/ml). Leukocytes were co-cultured with cell lines immediately after isolation. Plasma was stored at −70 °C for subsequent virus detection. Occasional duodenal biopsies were collected in HBSS containing an antibiotic mixture (BD, Panta) for inactivating bacteria and fungi. Adherent cells were isolated upon mechanical dissection followed by digestion with collagenase IV and dispase I.

### Cell lines and virus strains

As shown in Table S2, human AV3, RD, HEL-299 and monkey LLC-MK2 cell lines^36^ were obtained from ECACC and cultured in DME/F12 medium with penicillin/gentamicin supplemented with 10% heat-inactivated FBS. XerumFree (serum substitute) was used for some experiments. Cell cultures were checked monthly for mycoplasma contamination (MycoAlert Plus Mycoplasma detection kit).

EV reference strains were obtained from ATCC or other sources (Table S2). pU57 DNA plasmids containing the entire 5′UTR region of four different EV types (CV-A6, CV-B3, PV-1, EV-D68) were outsourced and utilized for quantitative studies. RNA viruses not belonging to the EV genus [Parechovirus-3 (PeV-3), encephalomyocarditis virus-D (EMCV-D), HIV-1, HCV, measles, mumps, rubella] as well as human cell lines and leukocytes from a pool of blood donors were used to explore the possible cross-reactivity of primer pairs. Reference EV strains were propagated *in vitro*, titrated, and stored at −70 °C. For acute infection, subconfluent cultures were infected at multiplicity of infection 1 and incubated for 4–6 hours. Persistent infection was investigated in the above cell lines infected with EV isolates derived from human cases.

Multiple antiviral antibodies (Table S1) were used for indirect immunofluorescence of infected cells. Virus titration and neutralization assays for EV typing were performed as reported^63^. Antibody dilutions in DME/F12 medium were made in quadruplicate in 96-well flat bottom plates. Then, 100 cell culture infectious doses_50_ (CCID_50_) of virus were dispensed into each well and mixed with antibody dilutions or control medium. After 2 hr incubation at r.t. with rocking, 10^4^ cells were added to each well. CPE was read 5–7 days post-infection. Antibody titer was defined as the highest antibody dilution capable of preventing CPE.

Time-lapse microscopy: cell culture flasks were placed on a 36 °C incubator in air plus 5% (vol/vol) CO_2_ and imaged every 30 min for 72–160 hrs using a Diaphot-300 inverted microscope (10x; Nikon, Firenze, Italy). The Nikon NIS-D imaging software was used.

### Procedure for virus detection

The procedure is summarized in Fig. 1. Peripheral blood leukocytes or cells isolated from duodenal biopsies were co-cultured with four EV-susceptible cell lines (AV3, RD, HEL-299, LLC-MK2) in order to enrich for virus^36^. Blood leukocytes (or other live cell types) were chosen as preferred virus source since they could be washed free of antiviral antibodies before co-culture with cell lines. For each patient, 0.5 × 10^6^ leukocytes (granulocytes and mononuclear cells) or 10^5^ cells from biopsy samples were cultured on semi-confluent cell monolayers in T-25 flasks at 36 °C. Cultures underwent 3–6 serial passages over 12–20 days. In this way, passage by trypsinization could get rid of non-adherent cells. At the last passage, culture medium was collected from each culture. Supernatants derived from the different cell lines were mixed and used for RNA extraction. As a final step, AV3 or RD cells that had been co-cultured with the patients’ cells were grown in chamber slides and stained with antiviral antibodies.

Transmission of viral infection to uninfected cell lines through cell-free supernatant of infected cultures was demonstrated by incubating cell monolayers in T25 flasks with 1 ml of supernatant clarified by high speed centrifugation and passed through 220 nm or 100 nm filters.

### EV primer pairs

Oligonucleotide primers (Table S3) were designed using CLCbio Main Workbench 7.7 (Qiagen Bioinformatics, Aarhus, Denmark). The specificity of each primer was assessed using Primer-BLAST software (http://blast.ncbi.nlm.nih.gov/Blast.cgi). As reported^37^, partially conserved regions of the EV genome were chosen: 5′UTR (untranslated region), 2 C (membrane- and RNA-binding, helicase), 3Dpol (RNA-dependent RNA polymerase). For poliovirus type-1, -2, -3, primers to the VP1-coding region were devised. Published primer pairs to the 5′UTR region were also used^12, 39, 64^. pUC57 plasmids containing the whole 5′UTR sequence of CV-A6 (Hyogo9426 strain; A species), CV-B3 (Nancy strain; B species), PV-1 (Mahoney strain; C species), EV-D68 (SZ04/CHN/2015 strain; D species) where obtained from GenScript (Piscataway, NJ) and used to evaluate the sensitivity of PCR assays in terms of copy number/reaction. Twenty-six primer pairs (Table S3) were tested against select EV reference strains and clinical/environmental isolates belonging to the A, B, C, and D species in parallel with appropriate negative controls (Table S2). Positive results were confirmed by Sanger sequencing. Amplicons of viral isolates were identified based on best matches in public databases.

### RNA extraction, reverse transcription (RT), end-point PCR

For each patient, RNA was directly extracted from plasma and peripheral blood leukocytes. In addition, RNA was extracted from mixed cell supernatants of four cell lines^36^ that had been co-cultured with leukocytes of each case. Total RNA was extracted from 0.6 ml of sample with an automated m2000 sp instrument (Abbott Molecular) using magnetic nanoparticles. Nucleic acids were dissolved in 60 μl elution buffer. Extracted RNA was quantified with a NanoDrop 3300 fluorospectrometer using the RiboGreen dye diluted in TE buffer according to manufacturer’s instructions (Thermo Fisher Scientific, Monza, Italy). Veriti thermocyclers (Thermo Fisher Scientific-Applied Biosystems) were used for RT and PCR reactions. cDNA was produced from 12 μl of RNA input (50–150 ng per reaction) using SuperScript III RT or SuperScript IV RT with their own master mixes [containing proprietary RNase inhibitor and helper proteins, random hexamer primers or random hexamer primers plus oligo (dT)18 (42 °C, 60 min; final volume 20 μl)]. Platinum Taq Hot Start DNA polymerase and PCR master mix with 3 percent Platinum GC Enhancer was used according to manufacturer’s instructions (0.2 micromolar primers, 5 μl cDNA, final volume 50 μl). In PCR assays, primers were used with the following conditions: a) 95 °C, 10 minutes; b) 14 cycles of 95 °C, 30 seconds, 65 °C, 30 seconds (with 1 °C decrease at each cycle), 72 °C, 30 seconds; c) 30 cycles of 95 °C, 30 seconds, 51 °C, 30 seconds, 72 °C, 30 seconds.

Sensitivity of 5′UTR PCR assays was measured in terms of detectable copy number/ml using quantitated pUC57 plasmids containing the 5′UTR region of CV-A6, CV-B3, PV-1, or EV-D68. Serial ten-fold dilutions of plasmids were made in low-EDTA TE buffer. End-point PCR reactions were run in a final volume of 50 μl.

To avoid possible contamination, control EV genomes were not run in parallel with clinical samples. Sealed FlashGel 2.2% agarose cassettes were used as screening tool for PCR. Reactions deemed positive, were re-analyzed by electrophoresis in 1.5–3% agarose gels containing GelRed (Biotium; DBA, Segrate, Italy) and recorded with a gel documentation system (Gentaur, Bergamo, Italy).

To confirm sensitivity results obtained by end-point PCR, dilutions of pUC57-5UTR plasmids were analyzed by quantitative real time PCR using Brilliant II SYBR QPCR Master Mix with ROX in an Applied Biosystems 7500 PCR instrument (5 μl cDNA, final volume 50 μl, 42 cycles). Reactions were run in triplicate using no-template controls in each run. For select clinical samples (4 PPS and 4 T1D cases), the viral load was estimated as the threshold cycle (Ct), a correlate of the concentration of the target sequence. To determine if the increase in fluorescence was due to amplification of target, a dissociation profile was generated. PCR samples were subjected to a stepwise increase in temperature (55–95 °C) and fluorescence readings were collected at default temperature increments.

To replicate assay conditions utilized for clinical samples, a series of 10-fold dilutions of titrated EV isolates (CV-A16, CV-B4, PV-2, EV-94) were made in complete cell culture medium as a diluent. Total RNA was extracted from 0.6 ml of dilutions 10^−4^ to 10^−9^. Upon reverse transcription, each dilution was tested in duplicate by end-point PCR assays.

Commercial real-time PCR assays designed to detect EVs or polioviruses (“research use only” assays, Table S1) were run according to manufacturers’ protocols, including internal controls and virus controls (5 μl template cDNA, final volume 25 μl).

### Amplicon sequencing

Electrophoretic bands of interest were cut from 2–3 percent agarose gels and re-amplified by PCR with relevant primers (10–15 cycles). Re-amplified products were Sanger sequenced (ABI PRISM 3100 Genetic Analyzer; BigDye Terminator v1.1 cycle sequencing kit). Phylogenetic trees were constructed using the neighbor-joining method based on a comparison of PCR amplicon sequences with short homologous regions of published nucleotide sequences of EVs.

Unsuccessful attempts were made to perform direct next generation sequencing (NGS) by constructing Illumina libraries using cDNA prepared from the samples. The failure may be due to the extremely low quantities of virus-specific nucleic acids present together with the overwhelming amounts of host sequences^41^. Therefore NGS was used only to confirm sequences of PCR amplicons.

### Immunocytostaining

Uninfected and virus-infected cells were cultured in 4-well Millicell EZ Slides. Cell monolayers were fixed in 4 percent paraformaldehyde (PFA) in PBS (36 °C, 15 min), washed 3× in filtered PBS-1% FBS containing 0.05% NaN3, permeabilized with Triton X100 (0.05% in PBS; 15 min), washed 3× in PBS-1% FBS. An appropriate dilution of each antibody was incubated overnight at 4 °C in PBS-1% FBS. Indirect immunofluorescence (IIF) staining was obtained with the following secondary antibodies (Table S1): Alexa Fluor 488-goat anti-mouse IgG H + L, Alexa Fluor 488-goat anti-rabbit IgG H + L, FITC-goat anti-baboon IgG H/L, FITC-goat anti-human IgG (Fc region). Secondary antibodies were incubated for 2 hrs at 36 °C. Cell monolayers were counterstained with Evans Blue. ProLong antifade was used as mounting medium. Images were taken with a Nikon E80i (Nikon, Firenze, Italy) or a Leica TCS SP8 microscope (Leica, Milano, Italy).

### Bioinformatics and statistics

Nucleotide sequences were aligned and primers were designed with the help of: CLC bio Main Workbench 7.7 (Qiagen Bioinformatics, Aarhus, Denmark), Blastn and Primer-Blast (NCBI), Clustal Omega (EMBL), PrimerQuest (Integrated DNA Technologies, Leuven, Belgium), Oligoevaluator (Sigma-Aldrich), FigTree v1.4.3 (Andrew Rambaut, University of Edinburgh). Statistical analysis was performed using Prism (GraphPad Software, La Jolla, CA). Qualitative variables definite as percentage were compared using Fischer’s exact test or Pearson’s Chi-square test. Quantitative variables were compared using Mann-Whitney U test. A two-tailed P value less than 0.05 was considered significant. Adobe Photoshop CS6 and Illustrator CS6 were used for image processing and alignment.

## Electronic supplementary material

Supplementary Information
