# Supplementary material for: Revealing enterovirus infection in chronic human disorders: An integrated diagnostic approach
Source: Sci Rep. 2017 Jul 10;7:5013. doi: 10.1038/s41598-017-04993-y (PMC5504018; doi:10.1038/s41598-017-04993-y)
Supplement: Supplementary file 1 — Supplementary Information [file 41598_2017_4993_MOESM1_ESM.pdf]

(SREP-16-45023)

SUPPLEMENTARY INFORMATION

REVEALING ENTEROVIRUS INFECTION IN CHRONIC HUMAN DISORDERS:

AN INTEGRATED DIAGNOSTIC APPROACH

Angelo GENONI[1], Filippo CANDUCCI[1], Agostino ROSSI[1], Francesco BROCCOLO[2], Konstantin CHUMAKOV[3], Giorgio BONO[4], Jorge SALERNO-URIARTE[5], Alessandro SALVATONI[6], Alberto PUGLIESE[7], and Antonio TONIOLO[1]*

Supplementary Table S1. List of consumables, cell lines, culture media, molecular biology reagents, chemicals, antiviral antibodies, commercial diagnostic kits for enterovirus detection.

Supplementary Table S2. Virus strains, plasmids, human cell lines, leukocytes of blood donors utilized as controls.

Supplementary Table S3. Oligonucleotide primer pairs utilized in this study (5'-3').

Supplementary Table S4.
A) SYBR green PCR assay of quantitated pU57 DNA plasmids comprising the 5'UTR region of four different enteroviruses.
B) Enterovirus genome copy number/ml in medium of AV3 cell cultures infected with samples of selected PPS and T1D cases.

Supplementary Figure S1.
Uncropped images of 2.2% agarose gels represented in panel A of Figure 2 (end-point PCR with the 5UTR-Tok primer pair). Amplification of quantitated plasmids containing the 5’UTR region of CV-A6, CV-B3, PV1, EV-D68 enterovirus types. The first lane of each image contains a DNA ladder of the following composition (bottom up): 50bp, 100bp, 150bp, 200bp, 300bp, 500bp, 800bp, 1,500bp.

Supplementary Figure S2.
Uncropped images of 2.2% agarose gels represented in panel B of Figure 2 (end-point PCR with the 5UTR-C primer pair). Amplification of quantitated plasmids containing the 5’UTR region of CV-A6, CV-B3, PV1, EV-D68 enterovirus types.
The first lane of each image contains a DNA ladder of the following composition (bottom up): 50bp, 100bp, 150bp, 200bp, 300bp, 500bp, 800bp, 1,500bp.

Supplementary Table S1. List of consumables, cell lines, culture media, molecular biology reagents, chemicals, antiviral antibodies, commercial diagnostic kits for enterovirus detection.

Description	Purchased from
**Blood test tubes**	
Vacutainer K_2EDTA tube, Vacutainer K_2EDTA plasma preparation tube	BD (Milano, Italy)
**Plasticware**	
Flasks (T-25; T-75); 12-well and 6-well multiplates; cell scrapers; pipettes	Thermo Fisher Scientific-Corning (Monza, Italy)
Millex syringe filter units, PVDF pore size 0.45, 0.22, 0.10 µm; Millicell EZ 4-well glass slides	Merck-Millipore (Vimodrone, Italy)
**Cell culture**	
Cell lines: AV3; RD; HEL-299; LLC-MK2	European Collection of Authenticated Cell Cultures (ECACC, Salisbury, UK)
Fetal bovine serum (FBS); DME/F12 medium with Hepes; L-Glutamine; Pyruvate; Penicillin-Gentamicin; Hank's balanced salt solution (HBSS); Dulbecco Phosphate-buffered saline (DPBS); Trypsin-EDTA	LifeTechnologies-Gibco (Monza, Italy)
XerumFree (serum substitute)	TNC Bio (Eindhoven, The Netherlands)
Histopaque cell separation medium (density 1.077 and 1.119 g/ml); Bovine serum albumin (BSA); Bovine skin collagen solution; Collagenase type IV; Dispase-I	Sigma-Aldrich (Milano, Italy)
PANTA antibiotic mixture (Polymyxin B; Amphotericin B; Nalidixic Acid; Trimethoprin; Azlocillin)	BD (Milano, Italy)
MycoAlert Plus Mycoplasma Detection Kit; Accutase (cell detachment enzymes)	Euroclone-Lonza (Pero, Italy)
**Molecular biology**	
GoTaq DNA hot start DNA polymerase and master mix; DNA molecular weight markers; Tris-Acetate EDTA buffer (TAE); Agarose; High-resolution agarose	Promega Italia (Milano, Italy)
Reagents and disposables for the m2000sp automated instrument; DNA and RNA preparation kits; reagent vessels; deep well plates; disposable tips	Abbott Molecular (Rome, Italy)
Custom oligonucleotide primers; Water (PCR-grade); Tris-EDTA buffer pH 8.0 (TE); low-EDTA (0.1 mM) TE buffer pH 8.0; Elution (10 mM Tris-Cl) buffer EB pH 8.5	Sigma-Aldrich (Milano, Italy)
Superscript III and Superscript IV reverse transcriptase with VILO master mix [containing ribonuclease inhibitor, helper and stabilizer proteins, random hexamer primers OR mixture of random hexamer primers plus oligo (dT)18; dNTPs; $MgCl_2$]; Platinum Taq hot start DNA polymerase and PCR Master Mix; Platinum GC Enhancer	Thermo Fisher Scientific-Invitrogen (Monza, Italy)
Brilliant II SYBR QPCR Master Mix with ROX passive reference dye	Agilent Technologies (Cernusco sul Naviglio, Italy)
GelRed stain	DBA Italia-Biotium (Segrate, Italy)
FlashGel - DNA electrophoresis screening system	Euroclone-Lonza (Pero, Italy)
BigDye Terminator V1.1 Cycle Sequencing Kit; Centri-Spin purification columns	Thermo Fisher Scientific-AppliedBiosystems (Monza, Italy)
**Chemicals (molecular biology grade)**	
Ethanol; Isopropanol; Dimethyl sulfoxide (DMSO); N,N-Dimethylformamide; Acetone; Paraformaldehyde (PFA); Triton X100; Tween-20; Na-Azide	Sigma-Aldrich (Milano, Italy)
**Virus antibodies and indirect immunofluorescence**	
Mouse monoclonal antibodies (MAb): 9D5 (panenterovirus directed to the VP1 capsid protein); Coxsackie A9; Coxsackie A24 (cross reacting with Echovirus 34); Coxsackie B Blend; Coxsackie B1; Coxsackie B2; Coxsackie B3; Coxsackie B4; Coxsackie B5; Coxsackie B6; Echovirus Blend 4, 6, 9, 11, 30, 34 (cross reacting with Coxsackie A24); Echovirus 4; Echovirus 6; Echovirus 9; Echovirus 11; Echovirus 30; Enterovirus 70; Enterovirus 71 (cross reacting with Coxsackie A16); Poliovirus Blend; Poliovirus-1; Poliovirus-2; Poliovirus-3.	Merck-Millipore (Vimodrone, Italy)
Mouse MAb 5D-8.1 (panenterovirus directed to the VP1 capsid protein)	Dako (Milano, Italy)
Mouse MAb B324M (panenterovirus directed to the VP1 capsid protein)	Acris Antibodies (Herford, Germany)
Mouse MAbs 3D-02 and 3D-05 (panenterovirus directed to the 3Dpol enzyme)	Our own laboratory
Rabbit polyclonal antibody to Poliovirus-1, Poliovirus-2, Poliovirus-3; Human-Monkey MAb to Poliovirus 1 and 2	Konstantin Chumakov (FDA, Silver Spring, MD)
Alexa Fluor 488 goat anti-mouse IgG; FITC goat anti-Rabbit IgG; ProLong antifade; Evans Blue; DAPI	Thermo Fisher Scientific (Monza, Italy)
FITC goat anti-Baboon IgG; FITC goat anti-Human IgG (Fc region)	Antibodies Online (Aachen, Germany)
**Commercial research use only (RUO) real time RT-PCR assays (enterovirus and poliovirus)**	
[A] Coxsackie RT-PCR; [B] Poliovirus RT-PCR	DID-Liferiver (Milano, Italy)
[C] Enterovirus LC	Qiagen-Artus (Milano, Italy)
[D] Enterovirus Q-PCR	Nanogen (Trezzano, Italy)
[E] Enterovirus 1-RQ	Experteam (Venezia, Italy)
[F] Enterovirus RT-PCR; [G] Enterovirus-C RT-PCR; [H] Poliovirus RT-PCR	Sacace Biotech (Como, Italy)

Supplementary Table S2. Virus strains, plasmids, human cell lines, leukocytes of blood donors utilized as controls.

Virus / Human cells	EV Species / designation	Strain / Source
CV-A2	A	PR92 - University of Parma, Italy
CV-A6 Hyogo9426 (5'UTR pUC57 plasmid)	A	GenScript, Piscataway, NJ
CV-A16	A	PR87 - University of Parma, Italy
EV-A71	A	QCMD[a]
CV-A9	B	Griggs – ATCC[b]
CV-B1	B	Conn-1 – ATCC
CV-B2	B	Ohio-1 – ATCC
CV-B3	B	Nancy – ATCC
CV-B3 Nancy (5'UTR pUC57 plasmid)	B	GenScript
CV-B4	B	JBV – ATCC
CV-B5	B	Faulkner - ATCC
CV-B6	B	Schmitt – ATCC
Echo11	B	Gregory – ATCC
Echo16	B	QCMD
Echo30 RNA	B	FIN/10/E3867/RDA/A2164 – THL[c]
CV-A1 RNA	C	Argene[d]
CV-A19	C	PR98 - University of Parma, Italy
CV-A24	C	QCMD
EV-C109	C	HSR, Milano, Italy
PV-1	C	Chat-1 vaccine – ATCC
PV-1	C	Sabin-1 vaccine – ATCC
PV-1 RNA	C	FIN/10/E3481/L20B85432-3 – THL
PV-1 Mahoney (5'UTR pUC57 plasmid)	C	GenScript
PV-2	C	Sabin-2 vaccine – ATCC
PV-2 RNA	C	FIN/10/E3481/L20B85412-3 – THL
PV-3	C	Sabin-3 vaccine – ATCC
PV-3 RNA	C	FIN/10/E3856/RDA87330-1 – THL
EV-D68 RNA	D	Argene
EV-D68 SZ04/CHN/2015 (5'UTR pUC57 plasmid)	D	GenScript
EV-D94	D	E210 – THL
Parechovirus-3 (PEV-3)	quality control strain	QCMD
Encephalomyocarditis virus (EMCV)	D-clone	J-W Yoon - NIH, Bethesda, MD
HIV-1	lab strain	P1 - ISS, Roma, Italy
HCV	clinical sample	GT-1b - Microbiology, Varese, Italy
Measles, Mumps, Rubella	combined vaccine	GSK, Siena, Italy
HeLa cell line - total RNA	–	ECACC[e]
AV3 cell line - total RNA	–	ECACC
HEL-299 cell line - total RNA	–	ECACC
RD cell line - total RNA	–	ECACC
Blood donors (peripheral blood leukocytes)	–	Transfusion Medicine, Varese, Italy

a. Quality Control Molecular Diagnostics (QCMD), Glasgow, UK.

b. American Type Culture Collection (ATCC); LGC Standards, Sesto San Giovanni, Italy.

c. National Institute for Health and Welfare (THL); Helsinki, Finland (courtesy Dr. Merja Roivainen).

d. bioMérieux-Argene; Bagno a Ripoli, Italy.

e. European Collection of Authenticated Cell Cultures (ECACC); Salisbury, UK.

Supplementary Table S3. Oligonucleotide primer pairs utilized in this study (5'-3').

Genome region	Designation	Sense primer (Fwd)	Antisense primer (Rev)	Amplicon size (bp)
**Enterovirus primers**				
5'UTR	EV 5UTR-Nij[64]	TCCTCCGGCCCCTGA	AATTGTCACCATAAGCAGCCA	156-155
	EV 5UTR-Tok[39]	TCCTCCGGCCCCTGAATGCGGCTAATCC	GAAACACGGWCACCAAAGTASTCG[a]	119
	EV 5UTR-3760[12]	TGGCTGCGTTGGCGGCC	TAGCCGCATTCAGGGGCCGGA	112
	EV 5UTR-3758[12]	TTCCTCCGGCCCCTGAATG	TGAAACACGGGCACCGAAAGTAGT	122
	EV 5UTR-A	GTGTAGATCAGGTCGATGAGTCAC	ATTGTCACCATAAGCAGCCA	293-296
	EV 5UTR-B	GACCAAGCACTTCTGTTACCC	GTCACCATAAGCAGCCAATATA	436
	EV 5UTR-C	GGTGTGAAGAGCCTATTGAGC	GATTGTCACCATAAGCAGCCA	186-187
	EV 5UTR-D	TGGTCCAGGCTGCGTT	AACACGGACACCCAAAGTAGT	210-212
2C	EV 2C-1A	ACAGTTCAAGTCCAAATGCCGTAT	GGGTTTTGGCACAGGTCATC	210
	EV 2C-1B	TACAGTTCAAGTCCAAATGCCGT	CTGTACATGGAGATAACNTCNAT	430
	EV 2C-2	CATACAGTTCAAGAGCAAACACCGT	ATGTTGGGGTACTTGCTAG	1317-1320
	EV 2C-3	GTTGCGACCAACTTNATTGC	TTTTGGTTTAGGTCGTCCA	213
	EV 2C-4	CAGTTCAAGTCCAAATCTCGCAT	ACTGGTGTATAGAGTTCCTTTTTC	309
	EV 2C-5	AAGAGCAAACACCGTATTGAACCT	GGGGTTTTGGCACAGATCATCCAT	352
3Dpol	EV 3Dpol-A	ATGGTTGCTTACGGAGATGAT	TTGGCATAGTAGGATGAATCA	214
	EV 3Dpol-B	GCACTGGGCATCAAGAAGAG	CCCTTGTCTGCTGGTGTCAT	719
	EV 3Dpol-C	CAGGAATAATAACAGGTTCAGCAGT	AGTCTTTTCCTGCTTGGGCTA	444
	EV 3Dpol-D	GGAAGAGGCAGTGGANCATTATGTG	AATAATCTATAAAAGACGTTTGATGNGTGT	581
**Poliovirus primers**				
5'UTR	EV-C 5UTR	GGTGTGAAGAGCCTATTGAGC	GATTGTCACCATAAGCAGCCA	186-189
2C	PV 2C-2	CATACAGTTCAAGAGCAAACACCGT	ATATTGGGGTACTTGCTAG	1317
3Dpol	PVs 3Dpol	GCAATGGGAAAGAAGAAGAGAGA	GTGGTCTAAATCTATGCCCTTGTA	597
	PV2 3Dpol	TTTGGGGACAGGGTGGATTAT	CCGCCCTTGACACAGTATGTT	83
	PV3 3Dpol	AGGAATCCAGGGGTCGTCACT	CCGCCCTTGACACAGTATGTT	257
VP1	PV1-VP1-B	TGCGTGGCCATTATAACCGT	CTGGAGCTGTTCCGTAGGTG	319
	PV2-VP1-C	CCGACAAAGCGCGCCAGCAG	GTGAGCCGCGTGGGGTTGTG	473
	PV3-VP1-C	ACAACCAACCACCCGGGCACA	ATGGCACTGAGATTCGCGCCG	308

a. N = A+C+G+T; S = G+C; W = A+T.

Supplementary Table S4.

A) SYBR green PCR assay of quantitated pU57 DNA plasmids containing the 5'UTR region of four different enteroviruses[a].

B) Enterovirus genome copy number/ml in medium of AV3 cell cultures infected with samples of selected PPS and T1D cases[a].

A) SYBR green PCR assay of quantitated pU57 plasmids containing the 5'UTR region of enteroviruses[a]			
pU57-5UTR plasmids	Input copy number per PCR reaction	Ct value[b] (n=3)	
		Mean	SD
Control buffer without template[c]	0	>42,0	—
pU57 CV-A6	16,000	24.3	0.29
	1,600	28.4	0.35
	160	31.9	0.60
	16	36.4	1.28
	1.6	39.8	0.69
pU57 CV-B3	16,000	23.5	1.21
	1,600	27.8	2.51
	160	30.9	2.65
	16	35.4	4.46
	1.6	40.6	2.66
pU57 PV-1	16,000	24.9	1.21
	1,600	29.2	0.41
	160	33.8	2.24
	16	38.1	1.33
	1.6	40.4	4.21
pU57 EV-D68	16,000	24.5	0.86
	1,600	28.5	0.99
	160	32.4	0.89
	16	36.8	0.85
	1.6	40.0	3.39

B) Enterovirus genome copy number/ml in medium of AV3 cell cultures				
Samples from clinical cases	Ct value[b] (n=3)		Calculated viral genome copy number/ml	Mean viral genome copy number/ml
	Mean	SD		
Control medium of uninfected cell culture[d]	>42,0	—	—	—
PPS RBJ/va15	38.2	0.26	120	177
PPS GST/va11	37.0	0.18	230	
PPS CMP/va10	37.4	1.15	220	
PPS CCM/va11	38.8	2.01	140	
T1D MZA/va15	37.4	0.61	220	470
T1D BRJ/va10	36.1	5.73	440	
T1D NPD/va14	35.1	2.29	620	
T1D GRG/va12	35.3	0.51	600	

a. SYBR green PCR assay run for 42 cycles using enterovirus EV 5UTR-Tok[39] primer pair.

b. Detection of the 5'UTR enterovirus target is expressed as the threshold cycle (Ct), an inverse correlate of the target sequence copy number.

c. Tris-EDTA buffer (low-EDTA).

d. Serum-supplemented DME/F12 medium of uninfected AV3 cells.

Supplementary Figure S1.

Uncropped images of 2.2% agarose gels represented in panel A of Figure 2 (end-point PCR with the 5UTR-Tok primer pair). Amplification of quantitated plasmids containing the 5’UTR region of CV-A6, CV-B3, PV1, EV-D68 enterovirus types. The first lane of each image contains a DNA ladder of the following composition (bottom up): 50bp, 100bp, 150bp, 200bp, 300bp, 500bp, 800bp, 1,500bp.

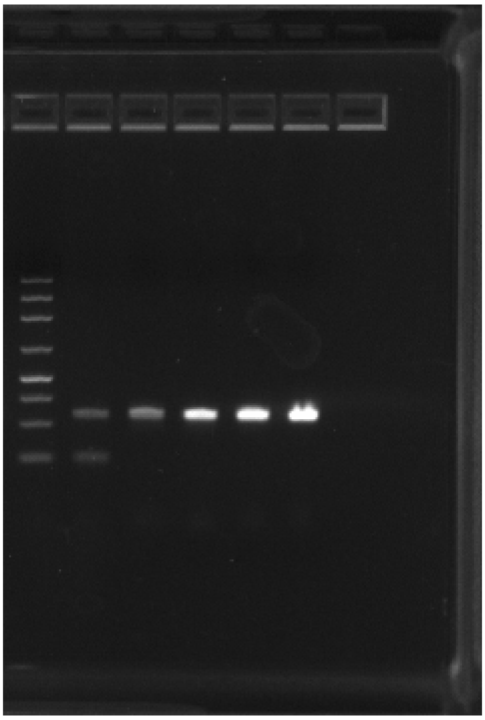

CV-A6

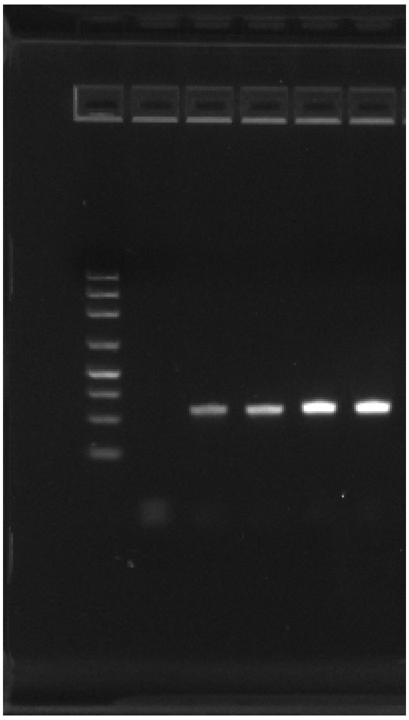

CV-B3

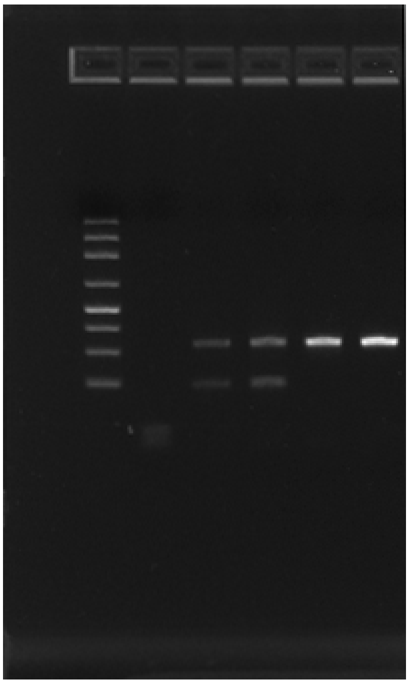

PV1

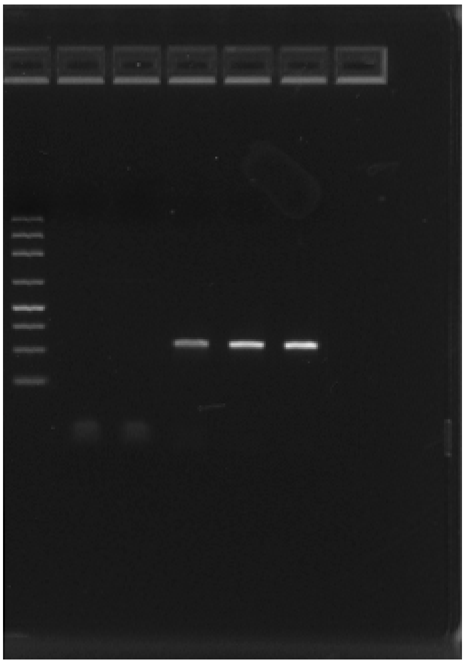

EV-D68

Supplementary Figure S2.

Uncropped images of 2.2% agarose gels represented in panel B of Figure 2 (end-point PCR with the 5UTR-C primer pair). Amplification of quantitated plasmids containing the 5'UTR region of CV-A6, CV-B3, PV1, EV-D68 enterovirus types.

The first lane of each image contains a DNA ladder of the following composition (bottom up): 50bp, 100bp, 150bp, 200bp, 300bp, 500bp, 800bp, 1,500bp.

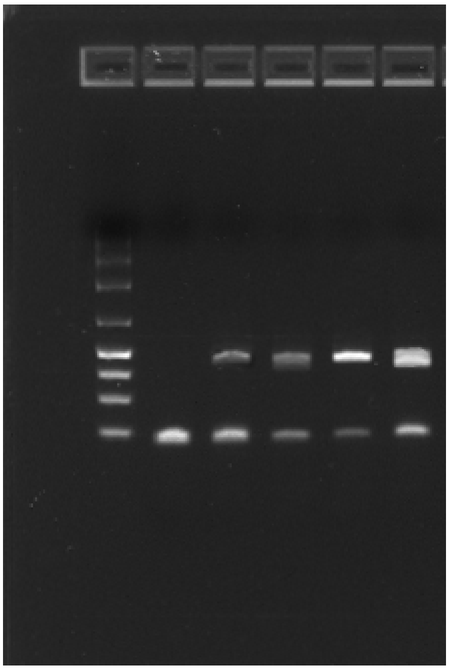

CV-A6

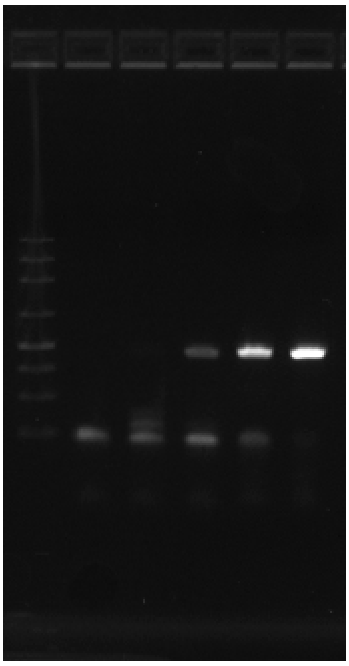

CV-B3

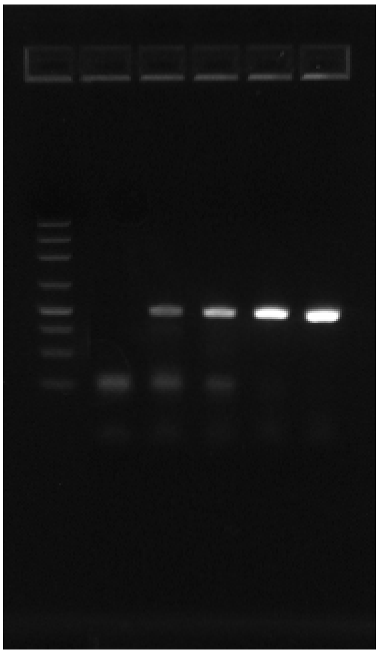

PV1

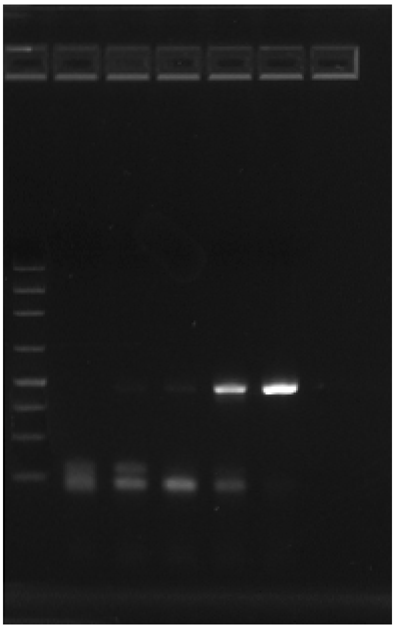

EV-D68
